# Augmenter of liver regeneration protects against carbon tetrachloride-induced liver injury by promoting autophagy in mice

**DOI:** 10.18632/oncotarget.14478

**Published:** 2017-01-03

**Authors:** Hongbo Shi, Weijia Han, Honglin Shi, Feng Ren, Dexi Chen, Yu Chen, Zhongping Duan

**Affiliations:** ^1^ Beijing Youan Hospital, Capital Medical University, Beijing, China; ^2^ Beijing Institute of Hepatology, Capital Medical University, Beijing, China

**Keywords:** augmenter of liver regeneration, autophagy, liver injury, apoptosis, proliferation, Pathology Section

## Abstract

**Background:**

Augmenter of liver regeneration (ALR) exerts strong hepatoprotective properties in various animal models of liver injury, but its protective mechanisms have not yet been explored. Autophagy is a recently recognized rudimentary cellular response to inflammation and injury. The aim of this study was to test the hypothesis that ALR may protect against acute liver injury through the autophagic pathway.

**Methods:**

The level and role of ALR in liver injury were studied in a mouse model of acute liver injury induced by carbon tetrachloride (CCl_4_). The effect of ALR on autophagy was analyzed *in vitro* and *in vivo*. After autophagy was inhibited by 3-methyladenine (3-MA), apoptosis and proliferation were detected in the mouse model with acute liver injury. The ALR and autophagic levels were measured in patients with liver cirrhosis (LC) and acute liver failure (ALF), respectively.

**Results:**

During the progression of acute liver injury, the ALR levels increased slightly in early stage and significantly decreased in late stage in mice Treatment with an ALR plasmid via tail vein injection protected mice against acute liver injury. The protective effect of ALR relied on the induction of autophagy, which was supported by the following evidence: (1) ALR overexpression directly induced autophagy flux in vitro and in vivo; and (2) ALR treatment suppressed apoptosis and promoted proliferation in mice exposed to CCl_4_, but the inhibition of autophagy reversed these effects. More importantly, the ALR levels decreased in patients with LC and ALF compared with normal controls.

**Conclusion:**

We demonstrated that ALR ameliorated liver injury via an autophagic mechanism, which indicates a potential therapeutic application for liver injury.

## INTRODUCTION

Acute liver injury is a liver function abnormality that results from multiple reasons, such as viral infection, abuse of drugs or alcohol, ingestion of toxic substance and so on [1, 2]. Serious or continuous liver injury often gives rise to liver cirrhosis or liver failure and results in death. The natures of acute liver injury have been deeply discussed [3], but the mechanisms of liver injury are still far from being clarified.

Augmenter of liver regeneration (ALR) is thought to be a hepatotrophic growth factor that is responsible for the extraordinary regenerative capacity of the mammalian liver [4]. A recent study found that unlike hepatocyte growth factor (HGF) or epidermal growth factor (EGF), ALR plays an important role in proliferation and anti-apoptosis in a liver-specific manner [5, 6]. Song et al found that ALR might protect against CCl4-induced liver injury by reducing mitochondrial dysfunction and inhibiting oxidative stress [7]. Gandhi et al found that liver-specific deletion of ALR caused hepatocellular necrosis, hepatic inflammation and fibrosis at 4-8 weeks after birth, which suggested that ALR is required for the development of liver [8]. Mu et al found that ALR treatment reduced pro-inflammatory cytokines, chemokines and iNOS in mice with concanavalin A (ConA)-induced hepatitis [9]. However, the protective mechanism of ALR in liver injury needs to be further explored.

Autophagy starts with the sequestration of a region of cytosol in double-membrane compartments followed by the formation of autophagosomes, the fusion of autophagosomes with lysosomes and degradation dependent on lysosome, which is regulated by autophagy-related genes (ATGs) [10, 11]. Autophagy represents an adaptive strategy by which cells can digest damaged organelles and provide energy for survival under stress and therefore autophagy has multiple roles in cell survival, proliferation and apoptosis in mammals [12, 13]. Our recent report has revealed that ALR protects HepG2 cells against apoptosis partly through an autophagic pathway [14].

Given the above information, we proposed that ALR may play a protective role in acute liver injury by regulating autophagic pathway. To test this hypothesis, we used a carbon tetrachloride (CCl_4_)-induced acute liver injury model to explore the protective mechanisms of ALR and measured the expression of ALR and the autophagic protein in patients with liver injury.

## RESULTS

### The expression profile of ALR in the progression of CCl_4_-induced acute liver injury in mice

**Figure 1:**
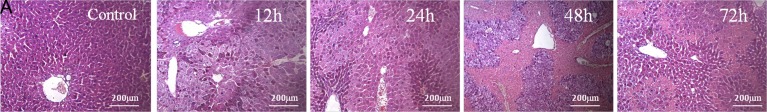

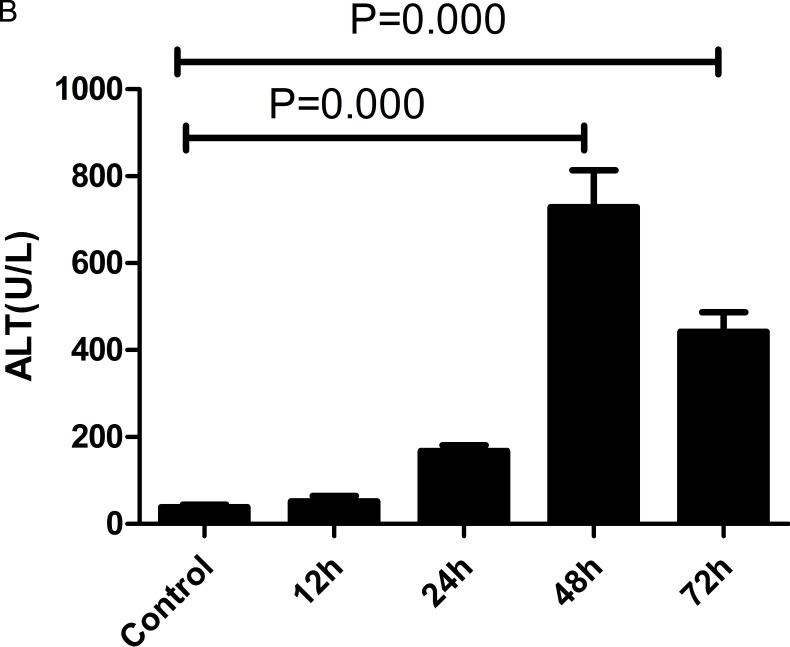

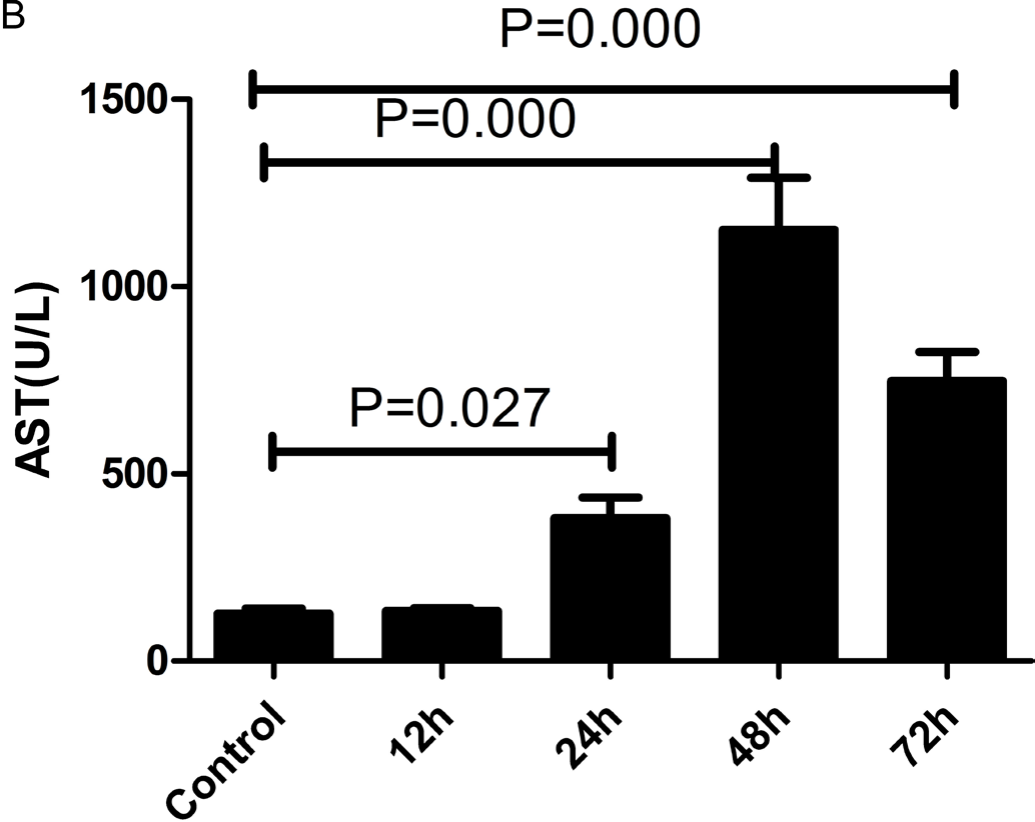

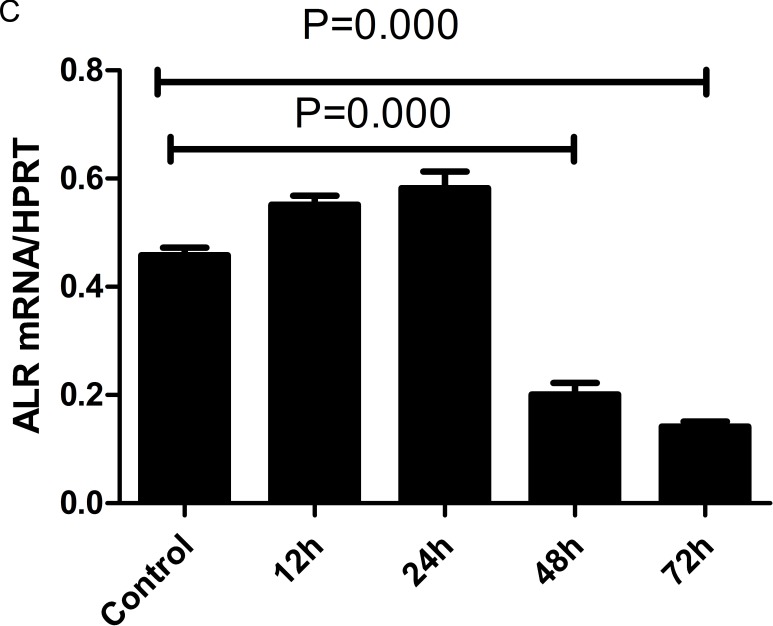

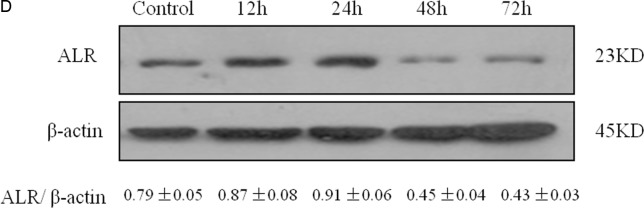
ALR expression is significantly decreased during the late stage in mice with CCl_4_-induced acute liver injury Mice received an intraperitoneal injection of a mixture of carbon tetrachloride (CCl_4_, 50%) and oil (50%) at a dose of 2 mL/kg body weight. The mice in the control group were injected with oil only. **A.** H&E staining of liver samples was performed from the control and 12, 24, 48, 72 h after the CCl_4_ injection. **B.** Serum AST and ALT levels were detected from the control and 12, 24, 48, 72 h after the CCl_4_ injection. **C.** Gene expression of ALR was measured by qRT-PCR in the livers of the control, 12, 24, 48, 72h after CCl_4_ injection. The average target gene/HPRT ratios for each experimental group were plotted. **D.** Protein expression of ALR was measured by western blot in the livers of the control and the 12, 24, 48, 72h after CCl_4_ injection. A representative blot from each group is shown. Densitometry analysis of the proteins was performed for each sample.

To investigate the role of ALR in acute liver injury, we examined the expression of ALR in mice exposed to CCl_4_. Bleeding and necrosis was apparent at 48 h after a CCl_4_ injection in the liver (Figure 1A). At the same time point, the levels of alanine aminotransferase (ALT) and aspartate aminotransferase (AST) in serum were increased (Figure 1B). Accompanying the liver injury, the ALR protein and mRNA levels displayed a slight up-regulation in the early stage (before 24 h) and a significant down-regulation in the late stage (after 48 h) after CCl_4_ injection (Figure 1C, 1D). These results indicated that ALR plays an important role in CCl_4_-induced acute liver injury.

### ALR protects mice against CCl_4_-induced acute liver injury

**Figure 2:**
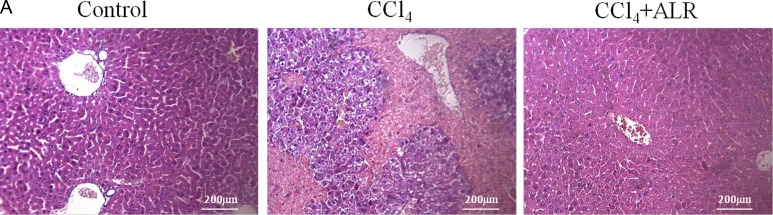

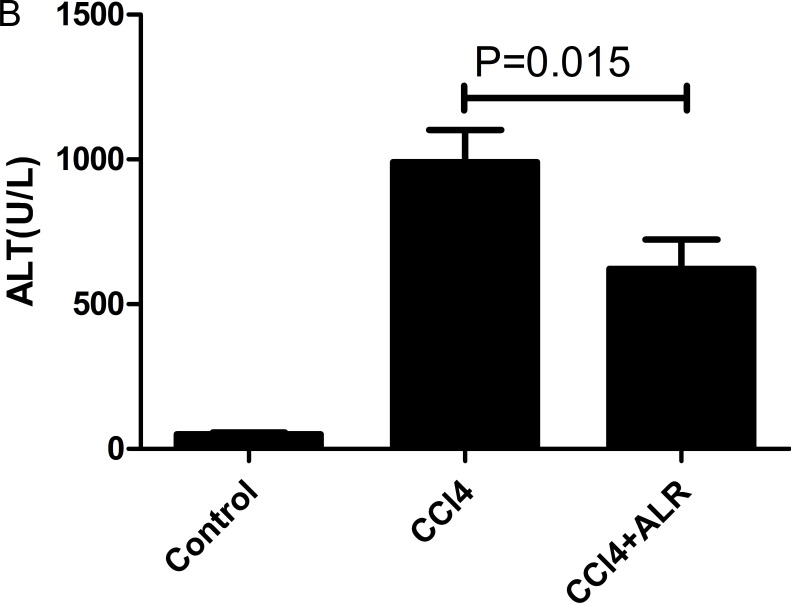

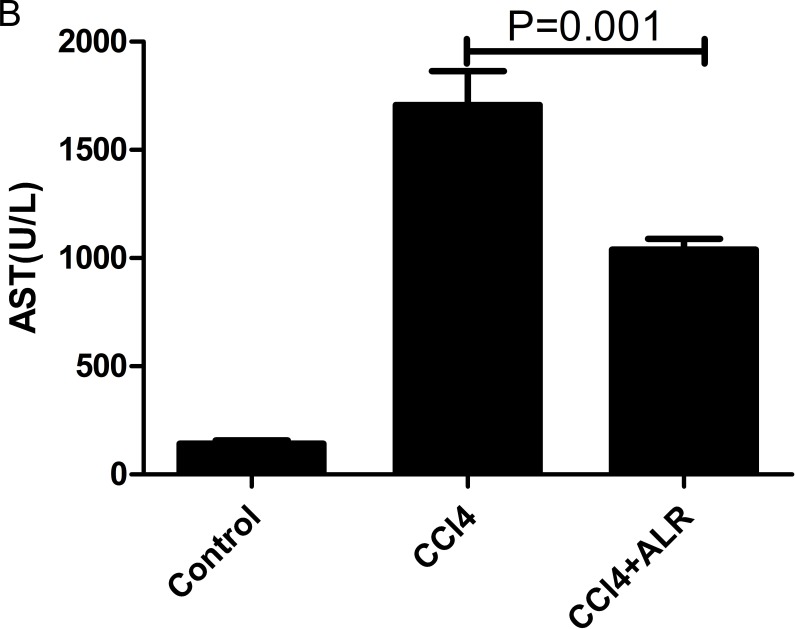
ALR protects mice against CCl_4_-induced acute liver injury The ALR plasmid (10 mg/kg) or pcDNA3.0 (10 mg/kg) was injected into the tail vein 6 h before CCl_4_ exposure. The mice were sacrificed 48 h after the CCl_4_ injection, and control mice were injected with oil only. **A**. H&E staining of livers was performed in control mice, CCl_4_-treated mice and ALR/CCl_4_-treated mice.**B**. Serum AST and ALT levels were measured in control mice, CCl_4_-treated mice and ALR/CCl_4_-treated mice.

We then evaluated whether ALR could play a protective role in mice with CCl_4_-induced acute liver injury. Treatment with ALR plasmids *via* a tail vein injection resulted in complete protection against acute liver injury. With respect to liver damage, the mice that had received the ALR plasmid treatment showed less bleeding and necrosis in liver tissue than those that had only been exposed to CCl_4_ (Figure 2A). In addition, the serum ALT and AST levels significantly decreased after ALR treatment (Figure 2B). These results demonstrated that ALR can protect mice against CCl_4_-induced acute liver injury.

### ALR promotes autophagy in CCl_4_-induced acute liver injury *in vitro*

**Figure 3:**
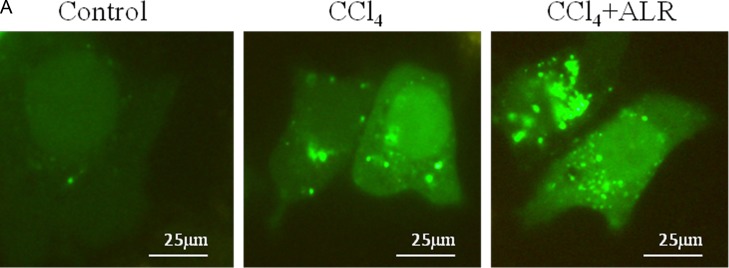

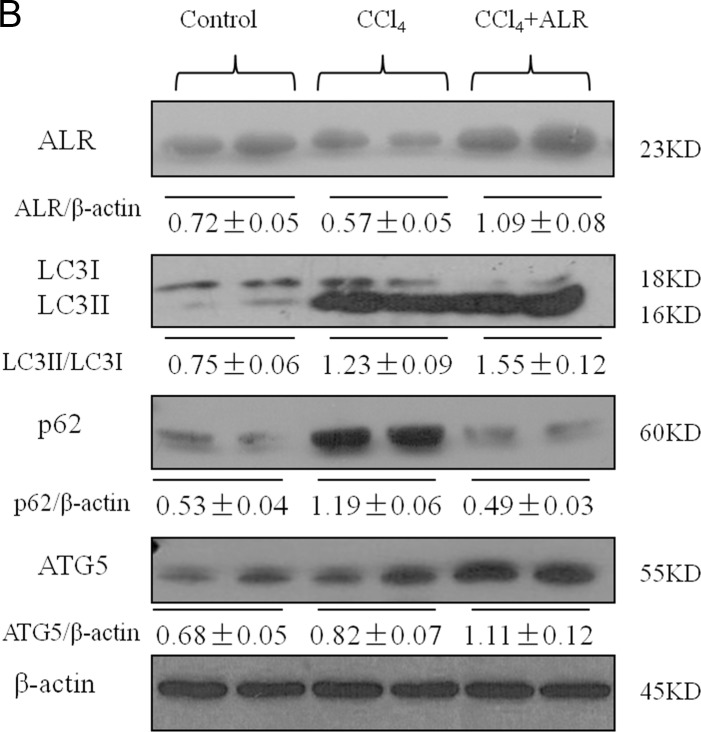
ALR promotes autophagy in CCl_4_-induced acute liver injury *in vitro* The ALR plasmid (1 μg/ml) or pcDNA 3.0 (1 μg/ml) was transfected into AML12 cells. Cells were then cultured in RPMI-1640 for 6 h, followed by treatment with CCl_4_ (10 mmol/L) for 48 h. The control cells were treated with DMSO only. **A**. GFP-LC3B plasmids (1 μg/ml) were transfected into AML12 cells to observe the formation of autophagosomes. The green puncta indicated the presence of autophagosomes in control cells, CCl_4_-treated cells and ALR/CCl_4_-treated cells. **B**. The expression of LC3B, Atg5, p62 and ALR was measured by western blotting in control cells, CCl_4_-treated cells and ALR/CCl_4_-treated cells. A representative blot for two samples from each group is shown. Densitometry analysis of the proteins was performed for each sample.

We next determined whether ALR could regulate autophagy. Microtubule-associated protein light chain three (LC3) and p62 have been widely used as markers of autophagy flux, in which the conversion of LC3I to LC3II indicates the closure of the autophagic vacuole and the p62 degradation indicates the fusion of autophagosome and lysosome [15]. GFP-LC3 behaves similarly to endogenous LC3 and displays punctate green fluorescence in autophagic formation [16]. AML12 cells, normal mouse hepatocytes, were cotransfected with ALR and GFP-LC3 plasmids to observe the effect of ALR on autophagy after CCl_4_ exposure. As shown in Figure 3A, green puncta in cells treated with ALR and CCl_4_ was more than those treated with CCl_4_ only. In addition, ALR treatment promoted LC3II conversion and p62 degradation and upregulated the ATG5 levels in cells exposed to CCl_4_ (Figure 3B). These results indicated that ALR could increase autophagy flux in hepatocytes exposed to CCl_4_.

### ALR promotes autophagy in CCl_4_-induced acute liver injury *in vivo*

**Figure 4:**
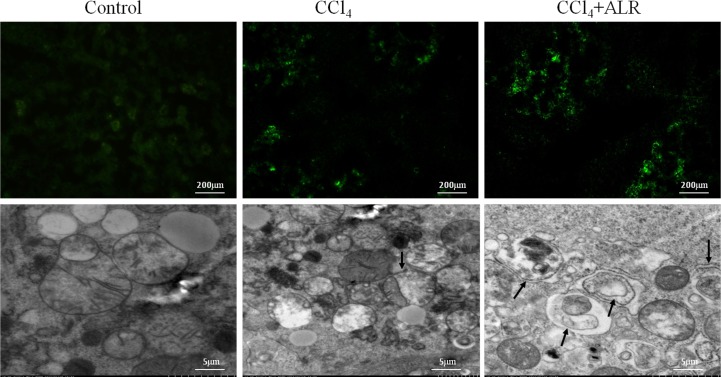

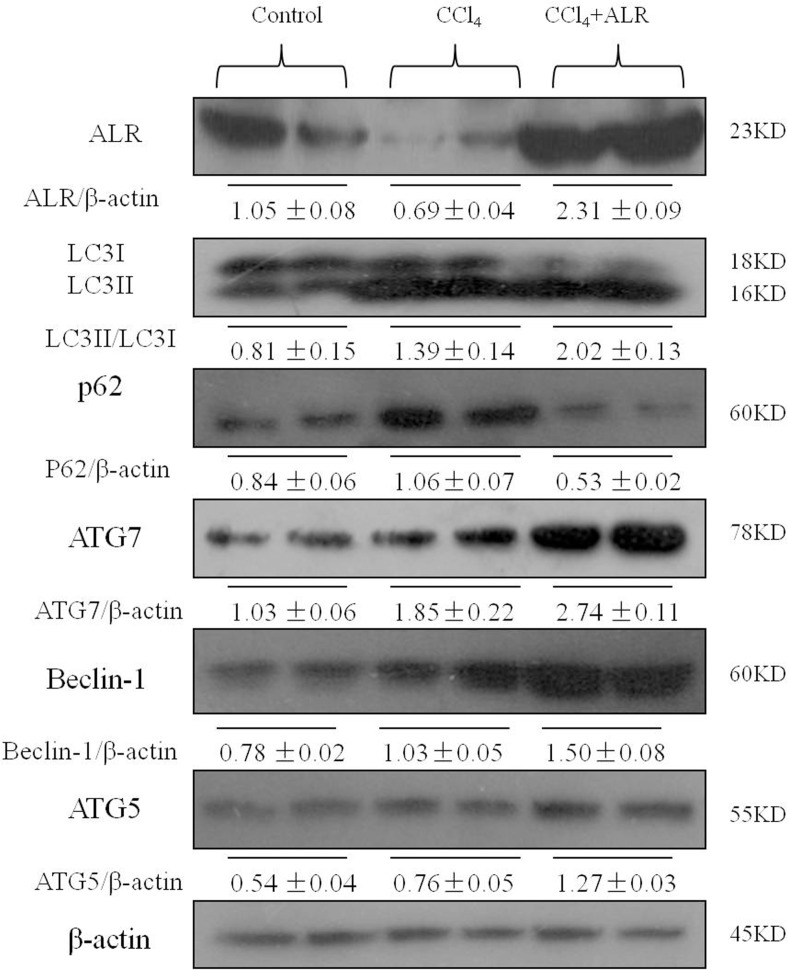
ALR promotes autophagy in mice with CCl_4_-induced acute liver injury The ALR plasmid (10 mg/kg) or pcDNA 3.0 (10 mg/kg) was injected into the tail vein 6 h before CCl_4_ exposure. The mice were sacrificed 48 h after CCl_4_ injection, and control mice were injected with oil only. The sections from control mice, CCl_4_-treated mice and ALR/CCl_4_-treated mice were observed in an inverted fluorescence microscope and an electron microscope. The expression of LC3B, Atg5, Atg7, Beclin-1, p62 and ALR was measured by western blotting in control mice, CCl_4_-treated mice and ALR/CCl_4_-treated mice. A representative blot for two samples from each group is shown. Densitometry analysis of the proteins was performed for each sample.

To further confirm our experimental findings *in vitro*, we then investigated the role of ALR on autophagy in mice that had been exposed to CCl_4_. As shown in Figure 4A, ALR treatment increased LC3 green fluorescence and the number of autophagosomes in the liver of CCl_4_-induced mice, which showed that ALR could increase autophagy *in vivo*. ALR treatment increased the expression of ATG5, ATG7 and Beclin-1 in mice exposed to CCl_4_. In addition, ALR treatment promoted LC3II conversion and p62 degradation in mice exposed to CCl_4_ (Figure 4B). These results indicated that ALR promoted autophagic flux in mice with CCl_4_-induced acute liver injury.

### ALR suppresses hepatocyte apoptosis in mice with CCl_4_-induced acute liver injury through an autophagic mechanism

**Figure 5:**
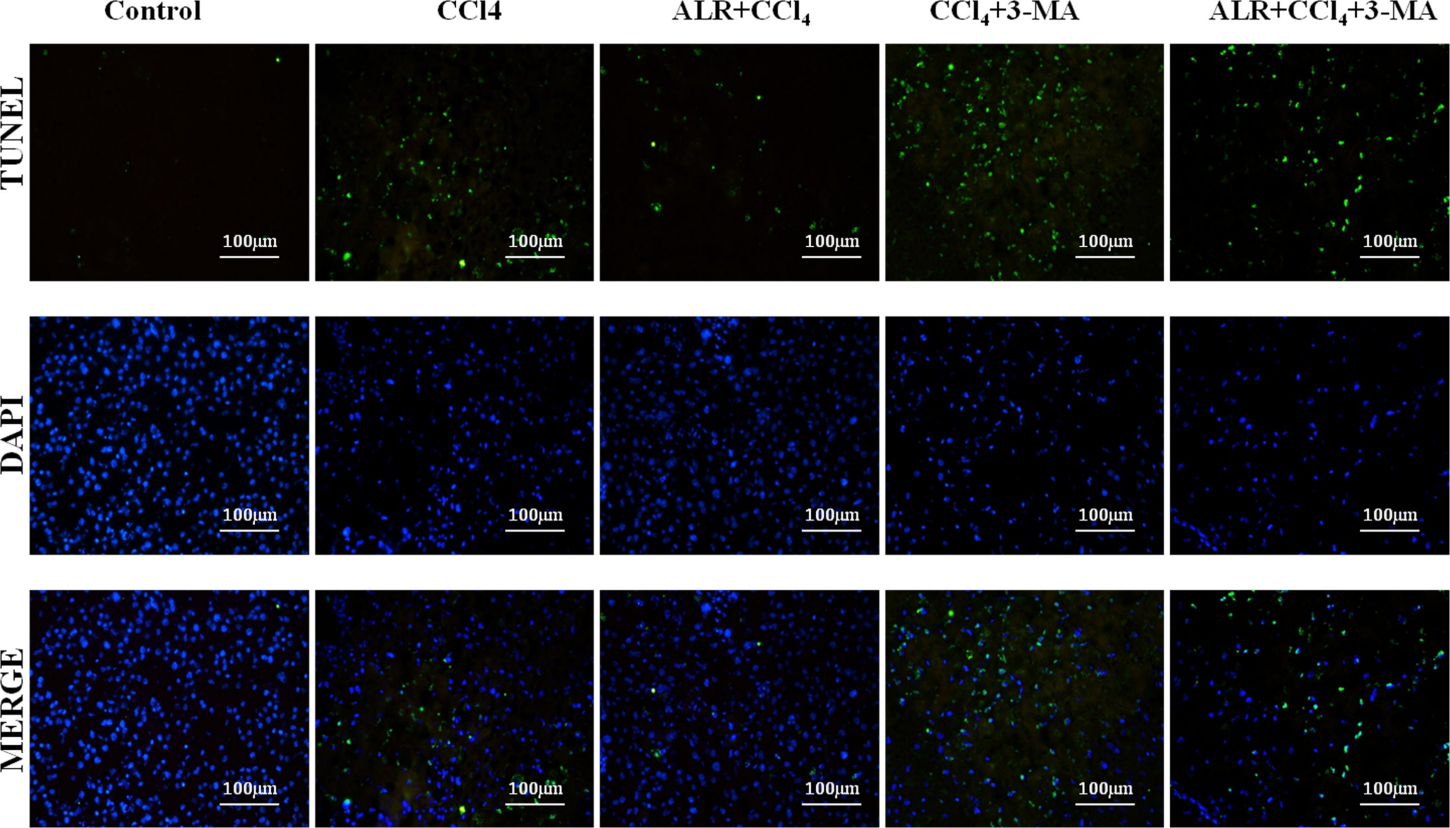
ALR suppresses apoptosis in mice with CCl_4_-induced acute liver injury through autophagic mechanisms The ALR plasmid (10 mg/kg) or pcDNA 3.0 (10 mg/kg) was injected into the tail vein 6 h before CCl_4_ exposure. The suppression of autophagy was achieved with a tail vein injection of 3-methyladenine (3-MA, 1 mg/kg) 2 h before CCl_4_ exposure. The mice were sacrificed 48 h after CCl_4_ injection, and the control mice were injected with oil only. Apoptotic cells were stained in control mice, CCl_4_-treated mice, ALR/CCl_4_-treated mice, CCl_4_/3-MA-treated mice and ALR/CCl_4_/3-MA-treated mice with a TUNEL apoptosis detection kit (KeyGEN BioTECH, Nanjing, China). All of the images were obtained on an inverted fluorescence microscope.

We then sought to investigate whether autophagy is required for hepatoprotective effect of ALR on acute liver injury. 3-methyladenine (3-MA), a class-III PI3 kinase (PI3K) inhibitor, was used to block autophagy because the complex of Beclin-1 and PI3K is required for autophagic vesicle nucleation [17]. As reported in a previous study [18–21], less apoptotic cells were observed in mice treated with CCl_4_ and ALR than those treated with CCl_4_ only, which suggested that ALR inhibits hepatocyte apoptosis. 3-MA addition significantly promoted cell apoptosis in mice treated with CCl_4_ and ALR, which indicated the inhibition of autophagy reverses the anti-apoptotic effect of ALR in acute liver injury (Figure 5). ALR was therefore able to suppress apoptosis through an autophagic mechanism in mice with CCl_4_-induced acute liver injury.

### ALR promotes hepatocyte proliferation in mice with CCl_4_-induced acute liver injury through an autophagic mechanism

**Figure 6:**
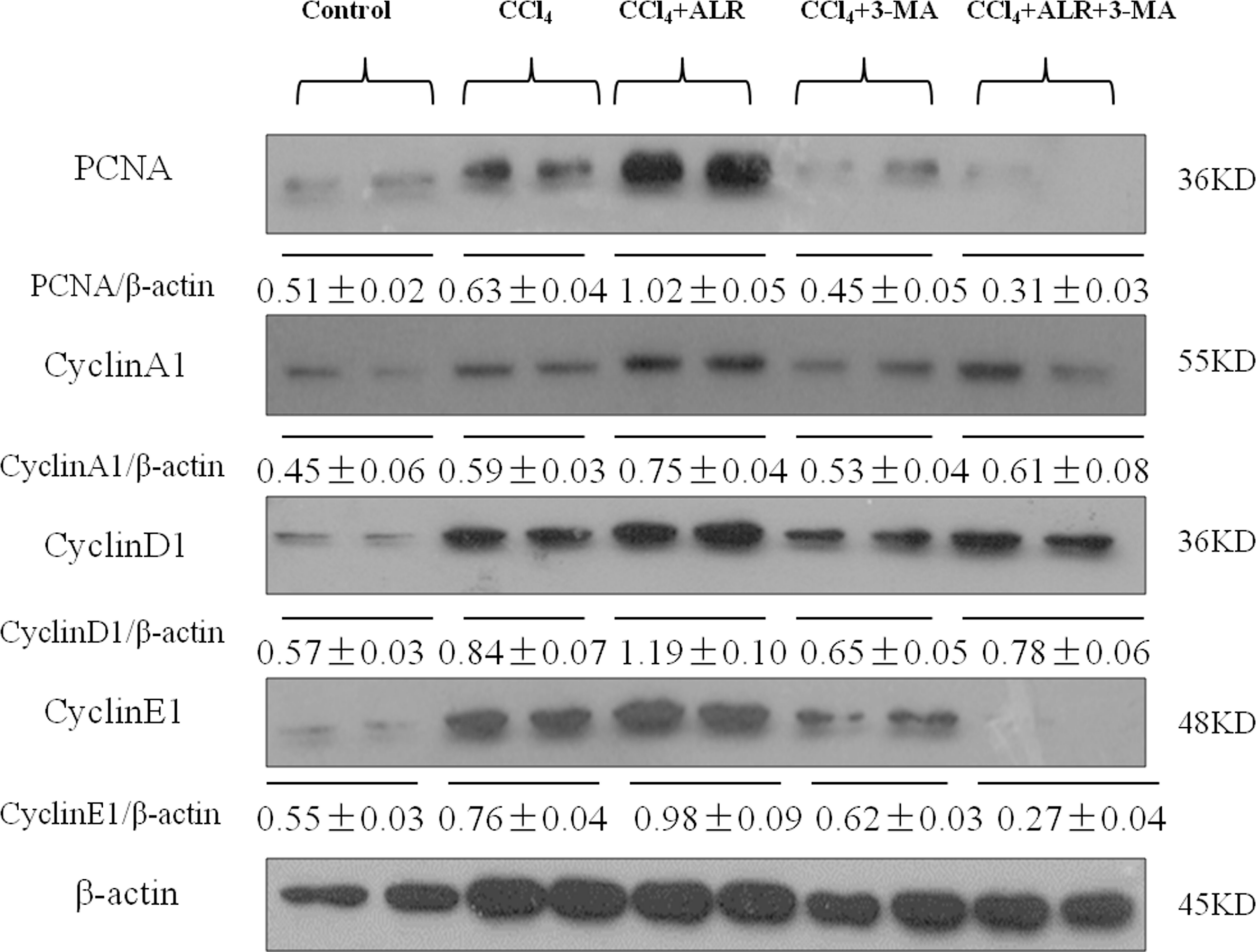
ALR promotes proliferation in mice with CCl_4_-induced acute liver injury through autophagic mechanisms The ALR plasmid (10 mg/kg) or pcDNA3.0 (10 mg/kg) was injected into tail vein 6 h before CCl_4_ exposure. Suppression of autophagy was achieved by tail vein injection of 3-methyladenine (3-MA, 1 mg/kg) 2 h before CCl_4_ exposure. The mice were sacrificed 48 h after CCl_4_ exposure, and the control mice were injected with oil only. The expression of proliferation-related proteins, including PCNA, CyclinA1, CyclinD1 and CyclinE1 was measured by western blotting in control mice, CCl_4_-treated mice, ALR/CCl_4_-treated mice, CCl_4_/3-MA-treated mice and ALR/CCl_4_/3-MA-treated mice. A representative blot for two samples from each group is shown. Densitometry analysis of the proteins was performed for each sample.

In addition to the anti-apoptotic effect, ALR has a role in cell proliferation and regeneration. Hepatic expression levels of cell-cycle associated proteins such as cyclins A, D and E were significantly higher in mice treated with ALR and CCl_4_ than those treated with CCl_4_ only, which suggested that ALR promotes DNA synthesis and mitosis. Moreover, the expression of proliferation cell nuclear antigen (PCNA) also revealed a similar trend as the cyclins. 3-MA addition significantly decreased the expression of PCNA and cyclins in mice treated with CCl_4_ and ALR, which indicated that the suppression of autophagy reverses the proliferative effect of ALR in acute liver injury (Figure 6). ALR was therefore able to promote hepatocyte proliferation through an autophagic mechanism in mice with CCl_4_-induced acute liver injury.

### The levels of ALR and autophagy in patients with LC and ALF

**Figure 7:**
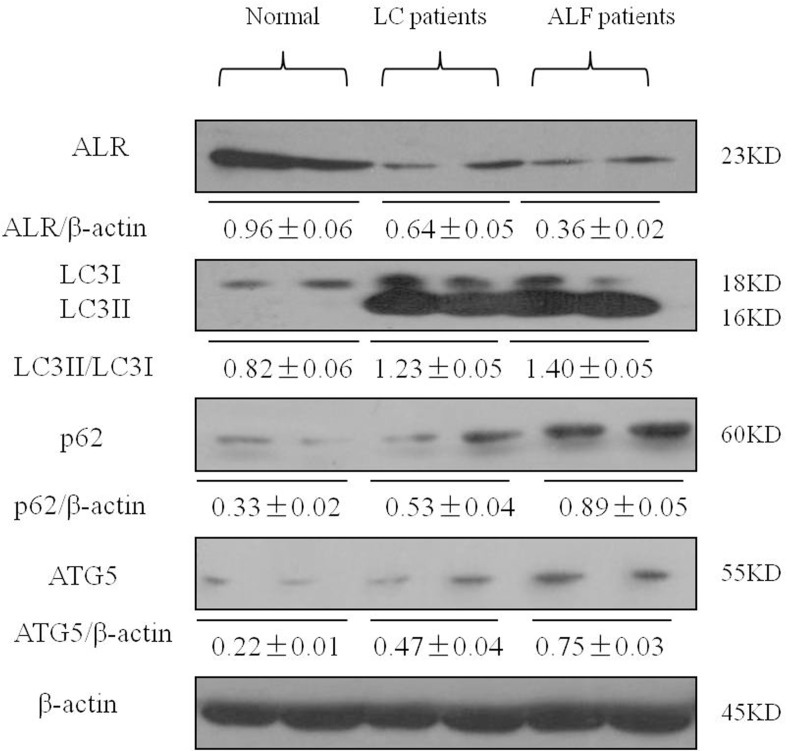
ALR expression is decreased in the liver of patients with LC and ALF The expression of LC3B, Atg5, p62 and ALR was measured by western blotting in normal controls and patients with LC and ALF. A representative blot for two samples from each group is shown. Densitometry analysis of the proteins was performed for each sample.

Finally, to determine whether ALR and autophagy participated in the disease process of liver injury, we measured the levels of ALR and autophagy markers in patients with liver cirrhosis (LC) and acute liver failure (ALF). Relative to the normal controls, LC3II conversion and p62 accumulation were significantly increased in patients with LC and ALF. ALR level was decreased in patients with LC and ALF compared to normal controls (Figure 7).

## DISCUSSION

Although ALR exerts strong hepatoprotective properties in various animal models of liver injury, its protective mechanism need to be further explored. The main findings of this study were that ALR promoted autophagic activity, which may have depressed hepatocyte apoptosis and increased hepatocyte proliferation, consequently resulting in liver protection.

ALR was discovered and isolated from the cytosol of neonatal rat liver and regeneration liver after partial hepatectomy [4]. ALR specifically stimulates DNA synthesis in hepatocytes and promotes recovery from liver injury, but its mechanism is unclear. Song et al found that ALR might protect against CCl4-induced liver injury [7]. Kumar et al found that compared to wild type mice, feeding alcohol to ALR-deficient mice resulted in hepatocellular injury and inflammation, ductular proliferation and fibrosis [22]. Similar to previous results [7], our results demonstrated that ALR could protect mice against CCl4-induced acute liver injury, which was mainly reflected in reduced bleeding and necrosis in liver tissue and low ALT and AST levels in sera after ALR treatment.

Some studies have shown that growth-factor-induced autophagy provides for cancer cells the important components of metabolism. Peng *et al* found that HGF increased LC3II conversion in primary mouse nonparenchymal cells (NPCs), and this effect was inhibited by an anti-HGF neutralization antibody [23]. Toshima *et al* found that HGF treatment increased LC3II levels in primary mouse hepatocytes [24]. These findings indicated that HGF promotes autophagy directly. ALR is similar to HGF in functions, so we speculated ALR may regulate an autophagy pathway as HGF.

The LC3II conversion and the p62 degradation are generally considered markers of autophagic flux [25]. Beclin-1, ATG5 and ATG7 take part in the initiation, extension and closure of autophagic vesicle respectively in the formation of autophagosomes [12]. Similar to previous results [14], our results demonstrated that ALR could upregulate ATG5, ATG7 and Beclin-1 expression and, more importantly, that ALR increased LC3II conversion and p62 degradation *in vivo* and *in vitro*. Collectively, our study revealed that ALR promoted autophagic flux in CCl_4_-induced acute liver injury. Interestingly, CCl_4_ exposure increased LC3II conversion and p62 accumulation, which suggests that autophagy may be induced during liver injury but that the fusion of autophagosomes with lysosomes may be blocked.

Increasing reports shows that autophagy has a protective effect on liver injury [26–28]. Ni et al found that autophagy protected hepatocytes against acetaminophen (APAP)-induced necrosis, whereas the treatment of 3-MA or CQ further exacerbated necrosis [27]. Ding *et al* also found that the inhibition of autophagy with CQ or siRNA significantly promoted hepatocyte apoptosis in an ethanol-induced model [28]. Toshima *et al* found that autophagic activity was increased in mice after partial hepatectomy (PHx), and the DNA synthesis and cell proliferation were impaired in Atg5 KO mice after PHx [24]. In our study, we found that ALR suppressed hepatocyte apoptosis but the inhibition of autophagy reversed the anti-apoptosis effect of ALR. In the same way, ALR promoted hepatocyte proliferation but the inhibition of autophagy reversed the proliferative effect of ALR. These results indicated that ALR could protect mice against CCl_4_-induced acute liver injury through an autophagic mechanism.

A previous study demonstrated that the levels of ALR in liver tissues were lower in patients with advanced alcoholic liver disease and nonalcoholic steatohepatitis than in controls [8]. Yu *et al* also found that ALR mRNA levels in patients with acute liver failure were decreased [29]. Similar to a previous study [29, 30], our study demonstrated that the level of ALR was decreased in patients with LC and ALF compared to normal controls. In addition, LC3II conversion and p62 accumulation were significantly higher in patients with LC and ALF than that in normal controls, which suggests that autophagic induction may be a response to liver injury and autophagic flux may be blocked.

Therefore, a clear understanding of how ALR influences autophagy can lead to the characterization of the underlying molecular mechanisms. Elucidation of the signaling cascades in ALR regulation and its mechanisms will be highly beneficial for the therapy and prevention of liver injury. In the future, a combined therapy regimen that includes ALR and autophagy may be a possible treatment strategy for patients with liver injury.

## MATERIALS AND METHODS

### Animal experiments

Male BALB/c mice (aged 6-8 weeks) were provided by the Animal Center at the Academy of Military Medical Sciences (Beijing, China). All of the animals were placed in a specific pathogen-free environment and received humane care according to the Capital Medical University Animal Care Committee guidelines.

The mice received an intraperitoneal injection of a mixture of carbon tetrachloride (CCl_4_, 50%) and oil (50%) at a dose of 2 mL/kg body weight. The normal control group received an intraperitoneal injection of the same volume of oil as the CCl_4_ group. The mice were scarified at 12 h, 24 h, 48 h and 72 h after the CCl_4_ injection. The ALR plasmid (10 mg/kg), which was a gift from professor An [31], was injected into the tail vein 6 h before CCl_4_ exposure, and a pcDNA3.0 (Invitrogen, NY, USA) as a control vector was injected into the tail vein at the same time. The suppression of autophagy was achieved with a tail vein injection of 3-methyladenine (3-MA, 1 mg/kg, Sigma) 2 h before CCl_4_ exposure. Serum aminotransferase was analyzed by the Clinic Center of Capital Medical University. H&E staining was performed by the Clinic Pathologic Center of Beijing Youan Hospital.

### Cell culture and treatment

AML12 cells, normal mouse hepatocytes (Jingkang Biological Company, Shanghai), were cultured in RPMI-1640 medium with 10% fetal calf serum in a 37°C incubator with 5% CO2. The GFP-LC3 and ALR plasmids were cotransfected into AML12 cells with the X-tremeGENE HP DNA transfection reagent (Roche, Shanghai, China) according to the manufacturer's instructions. A pcDNA3.0 as a control vector was transfected into AML12 cells. Cells were then treated with carbon tetrachloride (CCl4, 10 mmol/L) for 48 h to induce injury.

### Human specimens

The patients were recruited from the Artificial Liver Treatment and Training Center of Beijing Youan Hospital from 2013 to 2014. Normal liver samples were collected from five donors undergoing hepatic resection for liver transplantation. Liver samples with liver cirrhosis (LC) or acute liver failure (ALF) were obtained from the eighteen patients undergoing liver transplantation. Ten patients with LC were diagnosed according to the guidelines for the prevention and treatment of chronic hepatitis B (2010 version) [32]. Eight patients with ALF were diagnosed according to the guidelines for the diagnosis and treatment of liver failure (2012 version) [33].

### Fluorescence microscopy

After AML12 cells were transfected with GFP-LC3, GFP-LC3 puncta were observed in autophagic cells. To evaluate live tissues, the slides were incubated in an LC3 rabbit monoclonal antibody solution (Cell Signaling, CA, USA) overnight at 4°C and were then incubated in an Alexa Fluor 488 goat anti-rabbit IgG solution (Invitrogen, NY, USA) for 1 h at room temperature. Apoptotic cells were stained with a TUNEL apoptosis detection kit (KeyGEN BioTECH, Nanjing, China). All images were obtained with an inverted fluorescence microscope (Nikon Eclipse E800, Tokyo, Japan).

### Electron microscopy

Live tissues were fixed with 2.5% glutaraldehyde in 0.1 M phosphate buffer (pH 7.4). After dehydration, thin sections were cut and stained with uranyl acetate and lead citrate. Digital images were obtained with an electron microscope (H-7650, Tokyo, Japan).

### Western blot analysis

After the designated treatments were performed, liver tissues and cell pellets were lysed with RIPA buffer supplemented with protease inhibitors. Total proteins (50 μg) were separated *via* 12% SDS-polyacrylamide gel electrophoresis (PAGE) and transferred to polyvinylidene difluoride (PVDF) membranes. The membranes were incubated overnight with rabbit antibodies against ALR (Abcam, Cambridge, MA, USA), LC3B (Sigma, St. Louis, MO, USA), p62, Atg5, Atg7, Beclin-1, CyclinA, CyclinD, CyclinE, and β-actin primary antibodies (Cell Signaling, CA, USA) at 4°C. Then, the membranes were treated with a horseradish peroxidase-conjugated goat anti-rabbit secondary antibody (Cell Signaling, CA, USA) and developed with a chemiluminescent substrate (Thermo Fisher Scientific, Rockford, IL, USA). Densitometry analysis was performed using ImageJ software, and the relative levels of protein in each group were normalized to the loading control.

### Quantitative real-time polymerase chain reaction

Total RNA was extracted using TRIzol reagent (Invitrogen, NY, USA) according to the manufacturer's instructions. First-strand cDNA was synthesized from 5 μg of RNA (Superscript III cDNA Synthesis Kit, Invitrogen), and the ALR and HPRT mRNA levels were estimated by QPCR using a SYBR Green PCR Kit (Invitrogen, NY, USA) with a real-time PCR system (ABI PRISM 7300, MA, USA). The relative quantity of the cycle threshold value was normalized to an internal primer.

### Statistical analysis

All data are expressed as the mean±SD. One-way analysis of variance (ANOVA) followed by the *post hoc* LSD test was performed to compare differences among multiple groups. *P* < 0.05 was considered statistically significant. All data were analyzed using SPSS 11.5 software.

## Abbreviations

ALR: augmenter of liver regeneration
CCl_4_: carbon tetrachloride
3-MA: 3-methyladenine
LC: liver cirrhosis
ALF: acute liver failure
HGF: hepatocyte growth factor
EGF: epidermal growth factor
ATGs: autophagy-related genes
ALT: alanine aminotransferase
AST: aspartate aminotransferase
LC3: microtubule-associated protein light chain three
PCNA: proliferation cell nuclear antigen.
